# Can locally applied risedronate be an effective agent when combined with xenografts? An animal study

**DOI:** 10.1186/s12903-023-03231-4

**Published:** 2023-07-24

**Authors:** Taha Özer, Vusala Guliyeva, Alper Aktaş, Emre Barış, Mert Ocak

**Affiliations:** 1 Department of Oral and Maxillofacial Surgery, Hacettepe University, Ankara, Türkiye; 2 Department of Oral Pathology, Gazi University, Ankara, Türkiye; 3 Department of Anatomy, Ankara University, Ankara, Türkiye

**Keywords:** Bone transplantation, Bone histomorphometry, Risedronate, X-ray microtomography, Immunohistochemistry

## Abstract

**Background:**

To examine the effects of local risedronate application with xenografts on healing of rabbit skull defects using histological, histomorphometric, immunohistochemical, and three-dimensional radiological methods.

**Methods:**

Two critical-sized defects with a diameter of 10 mm were created in 16 rabbits and filled with xenogenic bone graft and xenogenic bone graft + 5 mg risedronate in the control I and risedronate (RIS) groups, respectively. Residual graft, new bone, soft tissue areas, and bone volume were evaluated in the 4- and 8-week study groups.

**Results:**

In both the 4- and 8-week samples, the RIS group samples had significantly higher mean new bone area values than the C group (p < 0.05). In both groups, the values for the new bone area were significantly higher in the 8-week-old samples than in the 4-week-old samples (p < 0.05). The h scores obtained for sialoprotein and osteopontin did not differ significantly between the groups at either time point (p > 0.05). The results of radiological evaluation showed that the bone density value was significantly higher in the C group than in the RIS group at either time point (p < 0.05).

**Conclusions:**

Although this study aimed to demonstrate the effect of risedronate on the osteoconductive properties of xenografts when applied locally, targeted results could not be achieved.

## Background

Although bone tissue is excellent for repairing fractures and cracks, there are some instances where this conventional mechanism is insufficient, and bone grafting procedures are required [1]. Grafting, a method widely employed in the repair of defects resulting from atrophy, injury, congenital malformations, tumor surgeries, or pathological degeneration, primarily aims to compensate for lost hard tissue [2]. Various natural and synthetic graft materials can be used for this purpose. As autogenous grafts contain osteogenic cells and contain osteoinductive factors that are essential for bone regeneration, they have long been considered the gold standard for bone replacement. However, increased morbidity due to the requirement of a second area of surgery, complications during and after surgery, limited availability, and anatomical limitations constitute the major disadvantages of autogenous bone grafts. Due to these disadvantages, different graft types have increasingly been used in clinical practice as alternatives to autogenous grafts, either by themselves or when strengthened with additional biochemical agents [3].

Allografts are grafts from living or dead human donors with few comorbidities. Although allografts are osteoconductive, their osteoinductive characteristics are limited by surface proteins inactivated by sterilization procedures. Additionally, they possess the risk of host rejection, despite having osteogenic potential. The use of allografts has been limited by the risk of contamination during processing, disease transfer from the donor, limited donor pool, and high processing and banking costs [4].

Xenografts are grafts of animal origin that can be obtained in higher volumes and at lower costs. They are generally of horse or cattle origin. As these grafts are obtained from different species, sterilization processes are more sensitive, further reducing the osteoinductive capacity of the graft. However, combining it with biochemical molecules is recommended to restore this lost osteoinductive effect [5, 6].

Risedronate (risedronate sodium) is a pyridinyl bisphosphonate that exhibits anti-resorptive activity when administered orally. The nitrogen atom in its cyclic structure allows for its administration at lower doses than other bisphosphonates, thereby increasing its potency. It inhibits the resorptive function of osteoclasts and increases the bone mass. Risedronate also has an indirect effect on osteoblast activity and bone formation, as demonstrated by reductions in serum osteocalcin and bone-specific alkaline phosphatase (ALP) levels. Risedronate, like other bisphosphonates, has a low oral absorption rate and is a hydrophilic molecule with a strong bone affinity. Risedronate inhibits dissolution in vitro by binding to hydroxyapatite crystals in the bone, is rapidly transported to the bone in vivo, and ultimately acts as an anti-resorptive drug. Additionally, it inhibits osteoclastic activity by inhibiting the farnesyl diphosphate synthase (FPPS) enzyme. This results in decreased bone turnover and resorption [7, 8].

This study aimed to examine the healing effects of local risedronate applied with xenogenic bone grafts on the defect areas created in the rabbit skull using histological, histomorphometric, immunohistochemical, and three-dimensional radiological methods.

## Methods

The study was performed according to the National Institutes of Health (NIH) ARRIVE guidelines for the care and use of laboratory animals and was independently reviewed and approved by the Animal Experiments Local Ethics Committee of Hacettepe University on September 24, 2019 (identification number 2019/10 − 01). In this study, 16 New Zealand male and female (50–50%) rabbits were used. One week prior to the study, the experimental animals were housed in cages under standardized room temperature, humidity, ventilation, and fluorescent light (12-hour night and 12-hour day) to adapt to environmental and climatic conditions. Two groups were designed for each experimental animal, with one defect on the right and the other on the left calvaria: Risedronate + Xenograft group (RIS) and Xenograft/control group (C).

Each group was divided into two healing time frames of 4 and 8 weeks for evaluation. In each experimental animal, it was ensured that the RIS group defect was on the left side of the calvaria and the C group defect was on the right side of the calvaria.

### Surgical procedure

All experimental animals received intramuscular ketamine hydrochloride (Alfamine, Alfasan, Netherlands) of 35 mg/kg and xylazine hydrochloride (Alfazyne, Alfasan, Netherlands) at 2.5 mg/kg for anesthesia. The right and left cranial areas were shaved, and the operation site was cleaned with povidone-iodine (Batticon, Adeka, Turkey). To control bleeding, infiltrative administration of 1 ml of local anesthetic solution (Ultracain D-S Forte, Sanofi Aventis, Turkey) was administered to the relevant area. The bone surface was exposed by making a full-thickness incision on the midline of the calvarium, including the periosteum ~ 4 cm in length, using a number 15 scalpel along the linea media. Using a trepan bur with an outer diameter of 10 mm and an inner diameter of 9 mm, two bone osteotomies were performed on the parietal bones, one on each side of the linea media without harming the dura, under sterile saline cooling.

- In the RIS group, 0.2 cc bovine xenograft (Cerabone, Botiss Biomaterials, Germany) with a particle size of 0.5–1 mm was first placed in 5 mg/ml risedronate (Sigma Aldrich) solution for 5 min and then placed on the defect area. The grafted area was then covered with a late resorbing collagen membrane (Collagen AT; Sistema AT, Padova, Italy) and rehydrated with a local risedronate solution.
- In the xenograft/control (C) group, each defect was grafted with 0.2 cc bovine xenograft (Cerabone, Botiss Biomaterials, Germany) with a particle size of 0.5–1 mm and covered with a late-resorbing collagen membrane (Collagene AT; Sistema AT, Padova, Italy).

**Fig. 1:**
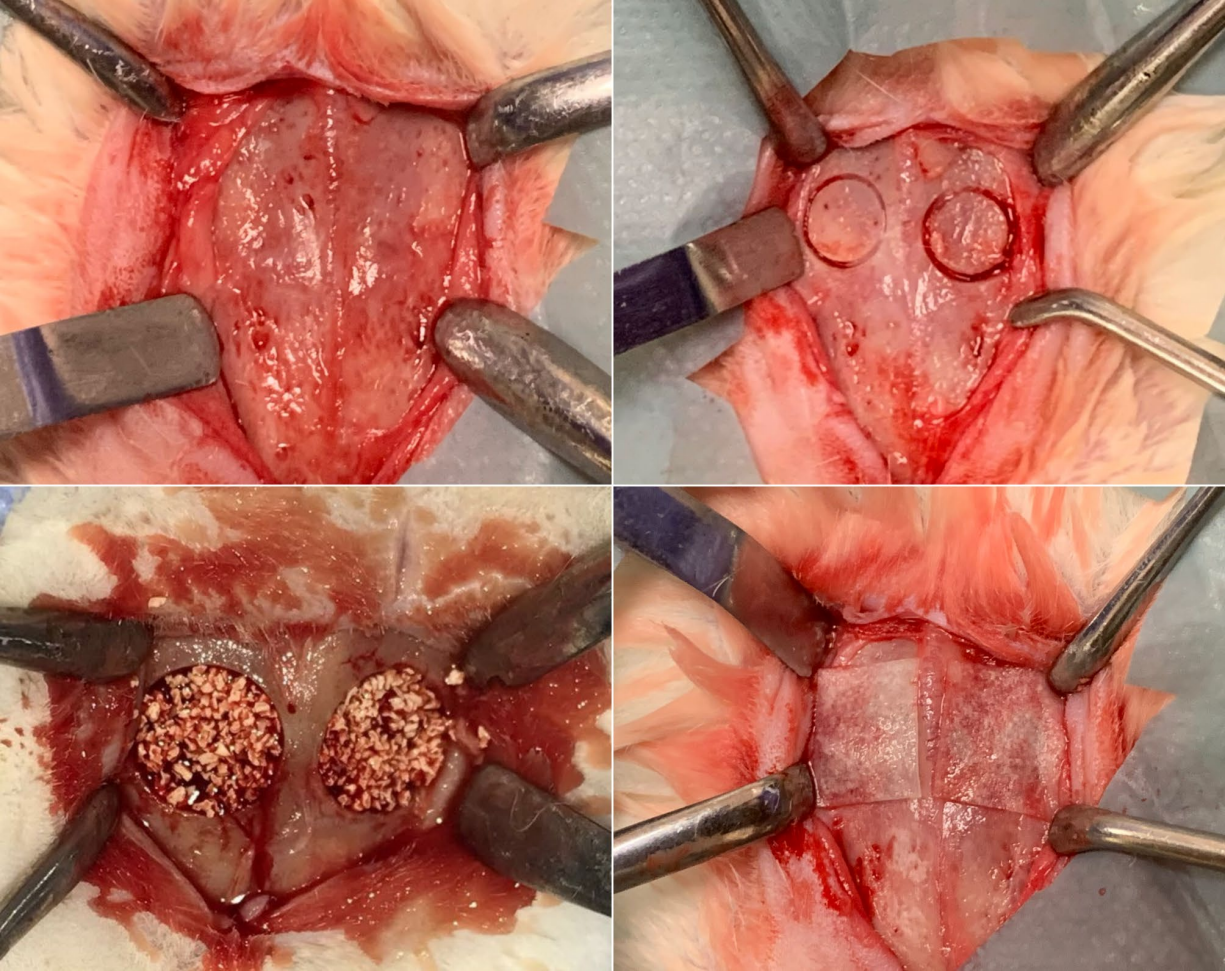
Protocol followed for bone grafting of calvarial bone defects in the rabbit model

Lastly, the skin and subcutaneous tissues were primarily sutured with a resorbable 16 mm 3/8 sharp 4.0 polyglactin suture (Coated Vicryl, Ethicon, Johnson & Johnson, Belgium). A wound dressing spray (Opsite, Smith, & Nephew, Canada) was applied to the sutured areas to prevent postoperative infections (Fig. 1).

In the postoperative period, 1 mg/kg of meloxicam (Maxicam X4, Sanovel, Turkey) as an analgesic and 2.5 mg/kg of enrofloxacin (Baytril-K 5%, Bayer, USA) as an antibiotic were administered intramuscularly to the animals once a day for 5 days. Each animal was kept in a separate cage for the duration of the experiment, with 12 h of light and 12 h of darkness. The ambient temperature was maintained at 22–24 °C on average, and the humidity was 55–70%. Regular health checks were performed on the wound areas and sufficient feed and water were provided.

### Tissue processing

Half of the experimental animals were sacrificed at the end of week 4 and the other half at the end of week 8 by intramuscular administration of lethal doses of xylazine HCl (30 mg/kg; Alfazyne, Alfasan, Netherlands) and ketamine HCl (70 mg/kg; Alfamine, Alfasan, Netherlands). En-block removal of the study area from the cranium with surrounding bone tissue intact was performed on each rabbit. Samples were separated into subgroups for each rabbit and fixed in 10% buffered formaldehyde for 48 h.

### Radiological analysis

The specimens were scanned using a micro-CT scanner (Skyscan 1174, Skyscan, Kontich, Belgium) with a pixel size of 40 μm. The X-ray tube voltage was 50 kV, and the current was 800 µA. The exposure time was 2300 ms. The X-ray projections were obtained at 0.70° intervals with a scanning angular rotation of 180°. The manufacturer-provided Nrecon software (Nrecon version 1.6.9.4; Skyscan, Kontich, Belgium) was used to perform subsequent reconstructions of the raw data obtained in this scanning step. 8-bit grayscale images reconstructed using Nrecon were imported into Ctan (version 1,13,5,1, Skycan, Kontich Belgium). The total bone volume and bone density values were obtained from selected regions of interest (ROI).

### Histological analysis

A total of 16 calvaria samples with two scaffolds on each were fixed with 10% formaldehyde for 48–72 h, and then samples were decalcified with the DeCastroR solution (300 ml absolute ethanol, 50 g chloral hydrate, 670 ml distilled water, and 30 ml 70% nitric acid) for 20 ± 2 days. Histomorphometric analyses were performed using a specialized image analysis software (Leica Qwin plus V3; Leica Microsystems, Wetzlar, Germany). Five different images were obtained from each hematoxylin-eosin-stained section at 100× magnification. In each image, the areas of new bone trabeculae and soft tissue were calculated in µm^2^. Only the mean values based on the measurements of the five images were retained.

Immunohistochemical staining was performed using the Thermo Scientific Shandon Sequenza Immunostaining Center device (Thermo Shandon Limited, Runcorn, Cheshire, United Kingdom). Four-µm-thick sections were cut from paraffin blocks and placed on slides. After the sections were kept in an oven at 60 °C for 1 h, deparaffinization was performed by placing them in xylol for 3 × 5 min. Then, the slides were dehydrated by passing them through a series of decreasing concentrations of alcohol (100%, 96%, 80%, and 70%). For antigen unmasking, a 1/10 dilution of citrate buffer (PH6) was applied to the Thermo Scientific Shandon Sequenza Immunostaining Center device (Thermo Shandon Limited, Runcorn, Cheshire, UK). Slides were attached to the rack slots and cover plates in the immunohistochemical staining device. A 5-min wash was performed with phosphate-buffered saline (PBS).

Tissue sections were treated for 10 min with 3% hydrogen peroxide (TA-125-HP; Thermo Scientific) to block endogenous peroxidase activity. Following the application of Protein Block (TA-125-PBQ; Thermo Scientific) for 5 min to the tissue sections washed with PBS, the sections were incubated with the primary antibodies anti-osteopontin (ab63856 Abcam) and Anti-Bone Sialoprotein (ab52128 Abcam) at + 4 ℃ overnight. The samples were then washed with PBS for 5 min and then kept in Amplifier Quanto (TL-125-QPB; Thermo Scientific) for 20 min and subsequently in HRP Polymer Quanto (TL-125-QPH; Thermo Scientific) for 30 min. PBS washing was performed at each stage. Staining was performed using DAB Chromogen (TA-125-HD; Thermo Scientific) to detect positive cells. Hematoxylin (HHS32; Sigma Aldrich) was applied for 30 s for background staining. Then, the samples were washed twice with distilled water for 1 min each, passed through a series of gradient alcohol (70%, 80%, 96%, and 100%), placed twice in xylol for 1 min each, and mounted with entellan (C1795 Merck).

Immunohistochemical analysis was performed under a light microscope at ×100 magnification. Staining in the extracellular matrix and cell cytoplasm within the defect area was considered positive for osteopontin (OSP) and bone sialoprotein (BSP). In OSP- and BSP-stained samples, staining intensity and extent were assessed jointly, and numerical data were obtained by following the h-score index (Table 1). The h-score was determined by multiplying the staining intensity by the staining intensity.

**Table 1:** h score index

Staining intensity		Staining extent	
0	No staining	0	No staining
1	Slight staining	1	% 0–25
2	Mild staining	2	% 26–50
3	Severe staining	3	% 51–75
		4	% 76–100
*** h*** = staining intensity X staining extent			

The h score index shows the staining intensity and its extent

### Statistical analysis

SPSS (version 21.0; SPSS Inc., Chicago, IL, USA) statistical package program was used to analyze the data. Descriptive statistics (mean, median, and standard deviation) were used in the analyses, and the Mann-Whitney U test was used for comparisons between different groups. In each group, data from weeks 4 and 8 were compared using the Wilcoxon test. Statistical significance was set at type 1 error level (alpha) values that were smaller than 0.05.

## Results

**Fig. 2:**
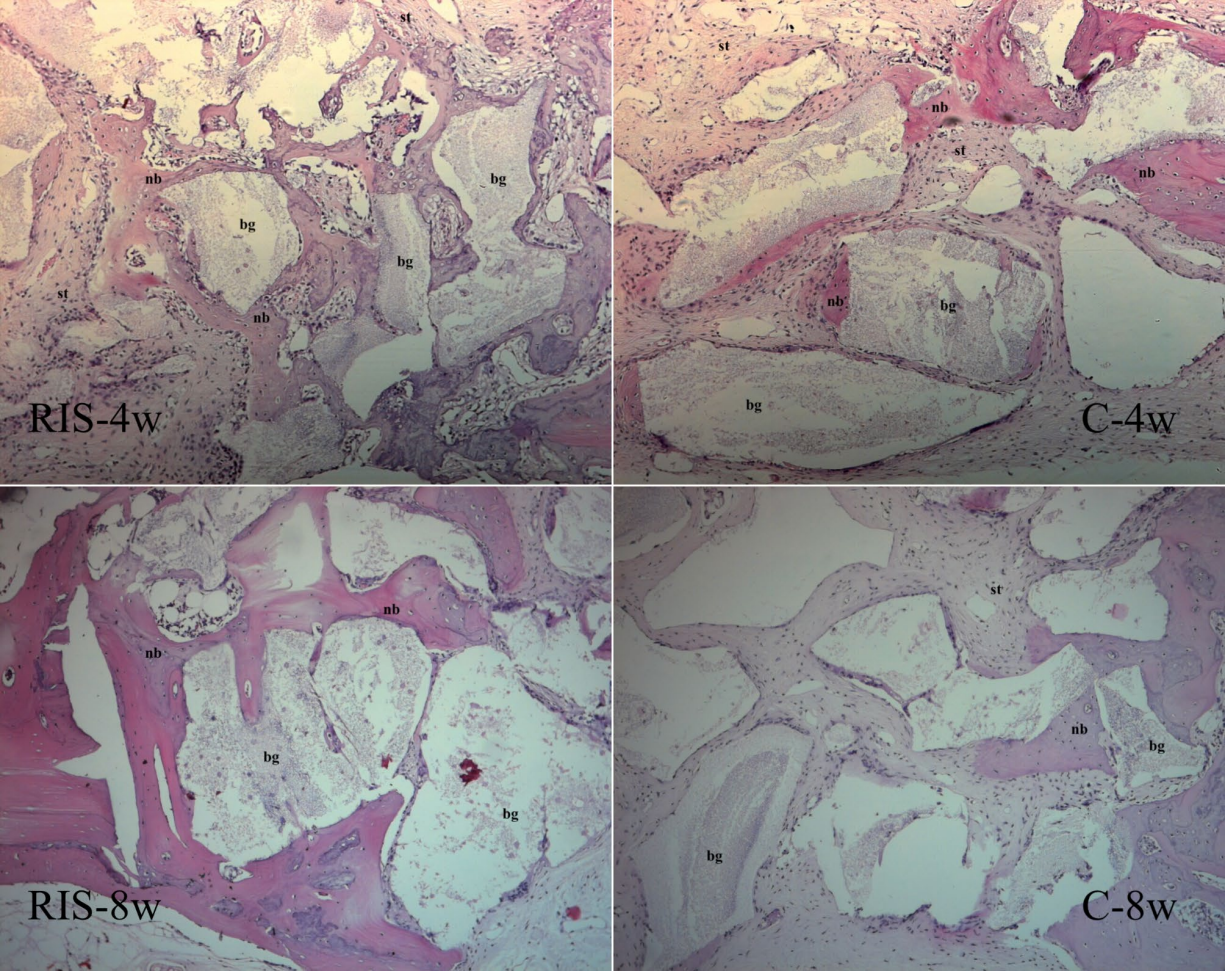
Rabbit model’s histological findings at 4 and 8 weeks post-surgery for calvarial bone defects. C group, xenogenic bone grafting only; RIS group, risedronate with xenogenic bone grafting; nb, new bone trabeculae; bg, bone graft material; st, soft tissue; hematoxylin and eosin, ×100 magnification

**Fig. 3:**
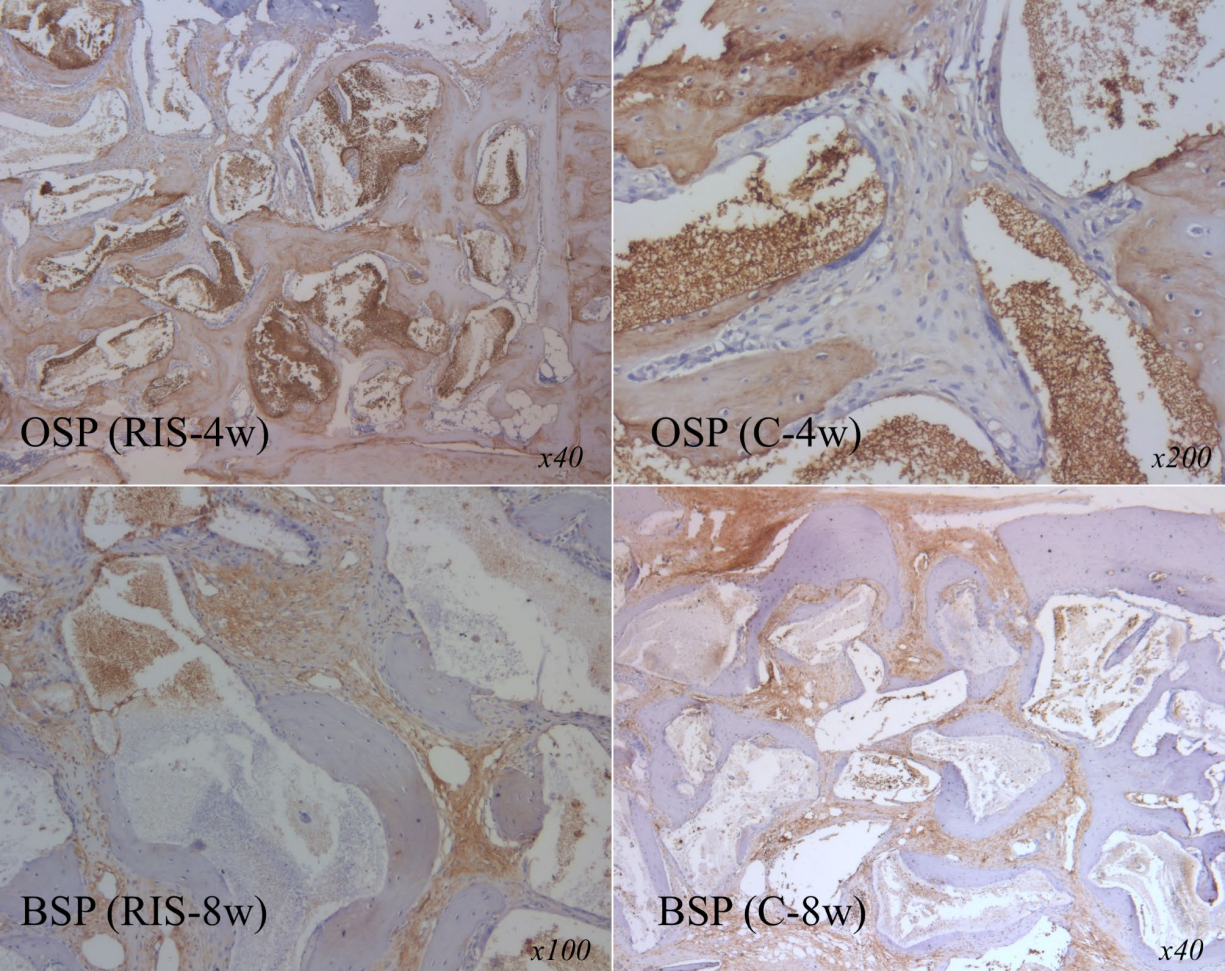
Immunohistochemical findings at 4 and 8 weeks post-surgery for calvarial bone defects in rabbits. BSP, bone sialoprotein; OSP, osteopontin; C group, xenogenic bone grafting only; RIS group, risedronate with xenogenic bone grafting

Examination of the histological preparations of the groups from week 4 showed that the defect area consisted of new bone trabeculae and loose collagenous connective tissue in almost all cases. It was noted that new bone production occurred from the edges toward the center of the defect. While new bone trabeculae were formed around the graft particles, most of them were localized at the periphery of the defect and had formed trabeculae because of reactivation of the injured periosteum. The general histological appearance of the week 8 histological preparations was similar to that of the week 4 samples from the same group, although there were noticeably more bone trabeculae, particularly in the RIS group. Fewer graft particles were present between the trabeculae in week 8 samples than in the samples collected from week 4 (Figs. 2 and 3).

In this study, histomorphometric analyzes (soft tissue area, new bone area, and residual graft area) were performed with data obtained from 2D histological sections. Radiological analyzes (total bone volume and BMD) were performed with data obtained from 3D micro-CT images.

**Fig. 4:**
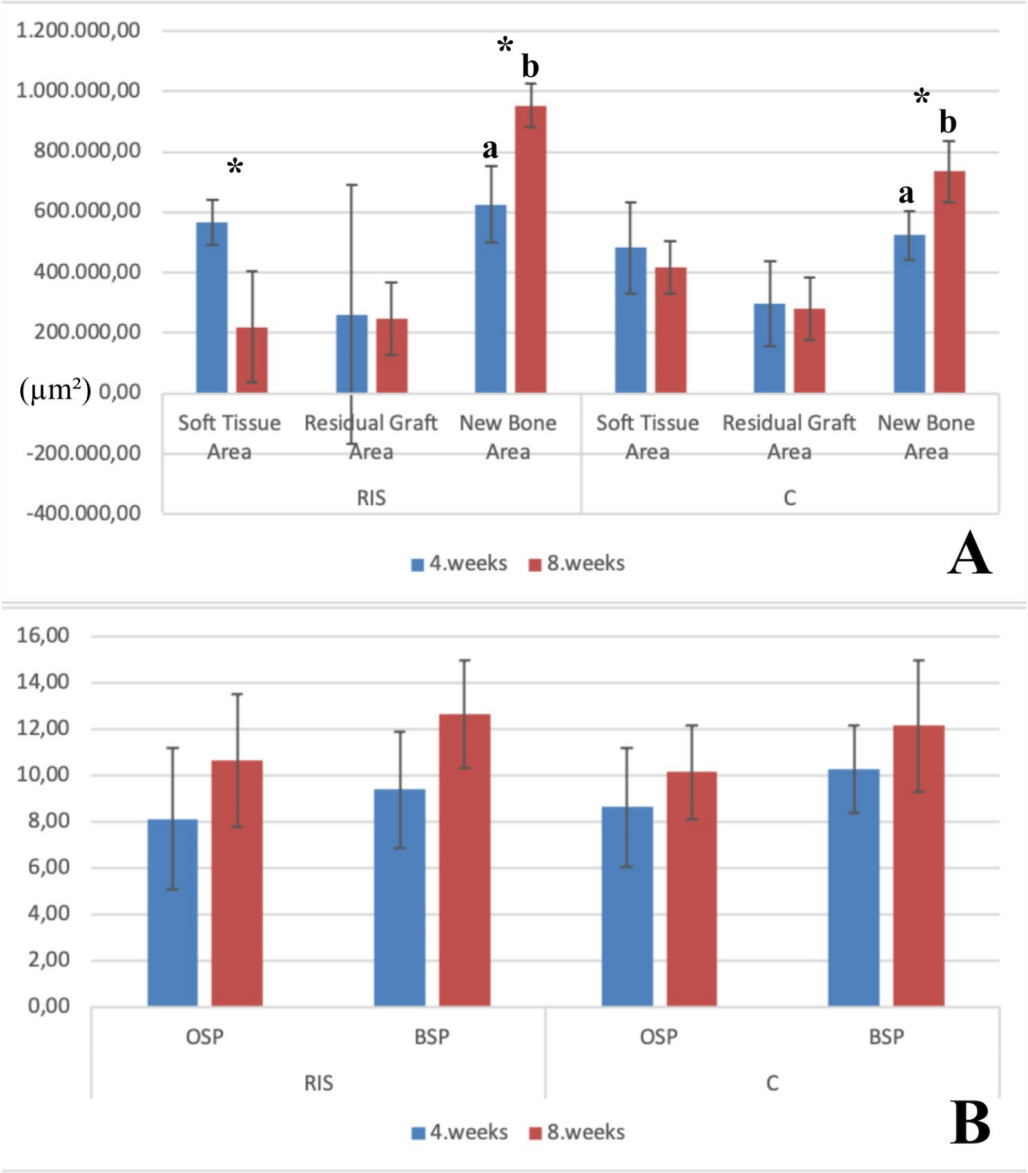
Statistical graphs of histological findings. (**A**). New bone, residual graft, and soft tissue area measured by histomorphometry at 4 and 8 weeks after surgery for calvarial bone defects in the rabbit model. Data are presented as the mean ± standard deviation. C group, xenogenic bone grafting only; RIS group, risedronate with xenogenic bone grafting; *, significantly different from values obtained 8 weeks after surgery; a, b, significantly different from values obtained in the other group; the black line indicates the standard deviation. (**B**). Comparison of h score values obtained from the immunohistochemical staining at 4 and 8 weeks after surgery. Data are presented as the mean ± standard deviation. C group, xenogenic bone grafting only; RIS group, risedronate with xenogenic bone grafting; the black line indicates the standard deviation

When the histomorphometric data from week 4 (soft tissue area, new bone area, and residual graft area) were evaluated, the new bone area value obtained in the RIS group was found significantly higher than that in the C group (p < 0.05). According to the results at week 8, the new bone area value of the RIS group was significantly higher than that of the C group (p < 0.05). The differences in the changes in histomorphometric parameters at weeks 4 and 8 in both the RIS and C groups were significantly different. While the new bone area values increased significantly from week 4 to week 8 in both groups (p < 0.05), the soft tissue area value decreased significantly in the RIS group (p < 0.05). Changes in the residual graft area were not significantly different between the groups (p > 0.05), (Fig. 4-A).

The h-score data determined from the BSP and OSP values based on immunohistochemical analyses did not show a significant difference between the groups at either week 4 or 8 (p > 0.05). Similarly, the increase from week 4 to week 8 in both the OSP and BSP groups was not significant (p > 0.05), (Fig. 4-B).

**Fig. 5:**
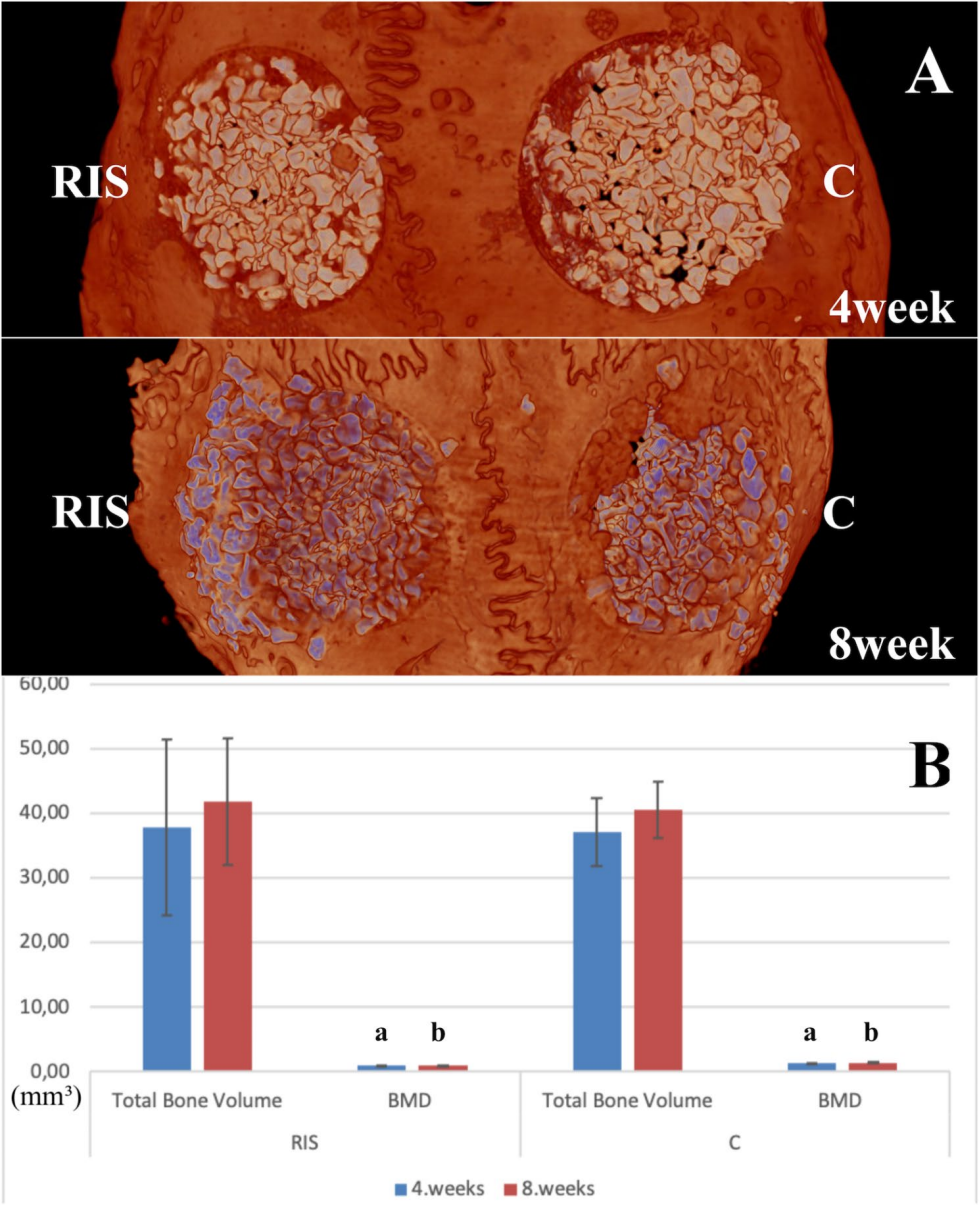
3-D radiological images and their statistical graphs. (**A**). Micro-computed tomography (CT) images of all groups at 4 and 8 weeks. (**B**). Comparison of values for the total bone volume and bone mineral density derived from radiographs obtained 4 and 8 weeks after surgery. Data are presented as the mean ± standard deviation. C group, xenogenic bone grafting only; RIS group, risedronate with xenogenic bone grafting; a, b, significantly different from values obtained in the other group; the black line indicates the standard deviation

There was no significant difference between the groups in terms of the total bone volume obtained radiologically at weeks 4 and 8 (p > 0.05). Additionally, the increase from week 4 to week 8 was not significant in either group (p > 0.05). The bone mineral density (BMD) of group C at week 4 and week 8 was significantly higher than that of the RIS group (p < 0.05), (Fig. 5).

## Discussion

This study aimed to examine the effects of risedronate, which was used locally with xenogeneic grafts for bone augmentation, on bone healing in terms of speed and bone quality, through immunochemical, histomorphometric, and radiological methods, and on its contribution to the acceleration of ossification after hard tissue grafting, which is frequently used in clinical applications.

Currently, indications for the use of bisphosphonates include postmenopausal osteoporosis in women, Paget’s disease, osteogenesis imperfecta, malignant hypercalcemia, and multiple myeloma. Despite extensive and effective treatment, systemic side effects such as renal toxicity, osteonecrosis of the jaw, atypical femur fractures, and hypocalcemia have recently been reported because of prolonged use of bisphosphonates [9, 10]. Moreover, it has been shown that a higher concentration level can be maintained in the targeted area [11, 12]. Toker et al. found no significant difference between the local and systemic applications of bisphosphonate in the defect area created in rat skull [13]. In a similar study by Küçük et al., no significant difference was found between the local and systemic application of bisphosphonates in a rabbit distraction model [14]. Local use of bisphosphonate-group drugs can have a positive effect in cases where bone healing is at the forefront due to the local suppression of osteoclastic activity. Using bisphosphonates locally, with minimal systemic side effects, is becoming more popular.

In a study by Jakobsen et al., the local dose of bisphosphonates was determined at 1 mg/ml [15]. Guimaraes et al. applied alendronate gel form at 10 mg/g locally in rabbits in their study [16]. In another study on dogs by Jakobsen et al., the local effect of alendronate was assessed by placing the allogeneic bone graft in a 2 mg/ml alendronate solution for 3 min and then placing it in the space created around the implant [17]. Cetinkaya et al. examined the effects of daily low-dose (0.1 mg/kg) and high-dose (1 mg/kg) oral risedronate on alveolar bone loss in mice with periodontitis. They concluded that short-term administration of low-dose risedronate inhibited bone resorption in mice, and high-dose and long-term administration led to impaired bone formation and angiogenesis [18]. Shabnam et al. used risedronate as a bisphosphonate-group drug and evaluated its effect on bone healing using histomorphometric analysis in their study of rabbits. They decided on 2 g of risedronate in 2% gel form as the application dose. When they compared the control group with the study group at the end of two months, they observed more ossification and osteoblast cells in the study group [19].

The present study used risedronate, a second-generation bisphosphonate group medicine, along with xenogenic bone graft particles and collagen membranes, to evaluate ossification using various parameters and evaluation methods. Risedronate was used locally in this study at a dose of 5 mg/mL and was shown to be effective in creating significant differences in new bone area and BMD. However, no significant differences were noted in the other parameters. One reason for this result may be the higher dose used in this study compared to that in other studies. Another reason may be the potential for risedronate diffusion between the adjacent defect areas.

Additionally, as there are insufficient studies examining whether biological agents administered locally can impact systemic circulation via interaction and this possibility can be listed as one of the study’s limitations. However, unlike other studies, risedronate was administered together with the xenografts in this study. The fact that this novel method did not yield positive results suggests possible interactions between risedronate and the xenograft. Further research is required to understand these interactions better.

Unlike the study conducted by Shabnam et al., in this study, risedronate was used in the pure form at a dose of 5 mg/ml and not in the gel form. This is significant for demonstrating the effects of risedronate and isolating the effects of additional biological materials. Khajuria et al. used biodegradable chitosan as a carrier for risedronate in the treatment of periodontitis in a rat model. Although positive results were reported, it is controversial whether chitosan could play a role in this effect [20]. Collagen membranes and graft materials were used as risedronate carriers in this study. In this method, xenografts and collagen membranes were maintained in a solution prepared with pure risedronate for 5 min. Consequently, the biological effects of the additional materials used as carriers were excluded.

In this study, the newly formed bone area was significantly larger in the samples from weeks 4 and 8 in the RIS group. This can be explained by the ability of risedronate to induce the production of new bone throughout each phase. This change, without changing the existing graft area, was supported by the significant reduction in the soft tissue area over time. When applied locally with grafts, bisphosphonates are expected to increase the total bone area by retaining the combined graft without resorbing it in the defect area. However, in this study, risedronate induced new bone formation without an anti-resorptive effect on the graft. Although this is attributed to the absence of viable cells in the xenografts, further research on the mechanism of local risedronate use is required.

Rabbits are considered suitable mammals because the hard and soft tissue healing response in the craniofacial region is similar to that in humans [21]. Rabbits weighing 3,500–4,000 g and aged 5–12 months were included in the study, as the skeletal growth of the rabbits included in the study should have been completed. The areas to create experimental defects in rabbits for research purposes were specified as the mandible, calvaria, femur, tibia, fibula, and radius. The calvarial bone was chosen as the area of interest in this study because of its ease of access and application, its ossification pattern, which is similar to that of the bones of the maxillofacial region, and its ease of comparison due to the availability of similar studies in the literature. Numerous successful studies have been published using rabbit skulls in the surgical field. Additionally, this region, with a sufficient surgical area, was selected to create both groups in the same animal and anatomical region to prevent individual differences.

A critical-sized defect is defined as the smallest bone wound in an animal that cannot heal spontaneously, with bone filling throughout life without using any osteopromotive material. For the rabbit skull bone, the size of the defect area was established as two circles with a 10-mm diameter. In these studies, bilateral calvarial defects with a 10-mm diameter were allowed to heal spontaneously, resulting in < 20% ossification [22, 23]. Working with defects of a critical size is valuable and necessary to showing the contribution of grafts or chemicals used in defects that cannot heal spontaneously to bone healing. In this study, critical-size defects determined for rabbit skulls were used.

The bone metabolism rate of experimental rabbits was determined to be three times faster than that of humans’ bone metabolism rate. Therefore, ossification that is completed within 6 months after surgery in humans can be evaluated as taking 8 weeks in rabbits [24]. Miloro et al. conducted studies with 2, 4, 8, and 12-week study groups to observe the early and late period bone healing status and showed that 4 and 8 weeks were the most appropriate evaluation times for early and late evaluation, respectively [25]. Therefore, the euthanasia times of rabbits were determined as 4 and 8 weeks to examine early and late ossification in our study.

Three-dimensional micro-CT and two-dimensional histomorphometric analyses were used to measure the amount of newly created bone and assess the soft tissue. Histomorphometric analyses require a long time for sample preparation and formation of artificial tissue and can only provide two-dimensional images [26, 27]. Therefore, current approaches include evaluating bone quality and quantity together with micro-CT instead of histomorphometric evaluation alone. Micro-CT has been considered both time-efficient and more suitable for examining bone microstructure, as it provides three-dimensional images in contrast to conventional two-dimensional histomorphometric analyses. The measurement of parameters, such as bone quantity and quality; bone volume and bone volume increase in the relevant region; bone volume fraction; and trabecular density, number, and parameters defining the degree of anisotropy, are examples of the morphometric advantages of micro-CT [28, 29]. Using this information as a reference, the degree of ossification and bone quality and quantity in rabbit calvaria were evaluated by histomorphometric and microcomputer analysis. Although the results obtained are generally compatible, it is important to ensure that the radiological data are based on the differences observed in some histomorphometric and radiological parameters. Here, the significant differences noted in the histomorphometric results concerning new bone area between the groups were not supported radiologically.

Moreover, immunohistochemical analysis is crucial for identifying important proteins in the extracellular matrix involved in bone formation. OSP and BSP were the proteins analyzed in this study using h-scores to evaluate the density and quantity of proteins in the samples [30]. The results showed no significant differences between the groups. However, the BSP and OSP levels increased in the 8-week compared to the 4-week samples. The increase in these protein levels over time in both groups was interpreted as an indication of strong ossification.

The limitations of this study include the ratio of drug dose and the method of administration, possible drug diffusion between defects, and the possibility of systemic effects of the locally applied drug. Furthermore, the co-administration of risedronate with viable autogenous grafts with osteogenic capacity rather than xenografts without viable cells will provide a more suitable environment for risedronate to take effect. Further research to eliminate these limitations is required to obtain more successful results.

## Conclusions

In conclusion, this study aimed to increase the osteoconductive properties of xenografts and to create better-quality bone in a short time by utilizing the local effect of risedronate, a bisphosphonate group drug; however, the targeted level of success was not achieved. Future studies should focus on determining the most appropriate dose of risedronate to be used as an effective agent when combined with xenograft.

## Data Availability

All data generated or analyzed during this study are included in this published article.

## List of Abbreviations

ALP: Alkaline phosphatase
BMD: Bone mineral density
BSP: Bone sialoprotein
C: Control
FPPS: Farnesyl diphosphate synthase
GBR: Guided bone regeneration
NIH: National Institutes of Health
OSP: Osteopontin
RIS: Risedronate
